# Interspecies introgressive hybridization in spiny frogs *Quasipaa* (Family Dicroglossidae) revealed by analyses on multiple mitochondrial and nuclear genes

**DOI:** 10.1002/ece3.3728

**Published:** 2017-12-21

**Authors:** Qi‐Peng Zhang, Wen‐Fang Hu, Ting‐Ting Zhou, Shen‐Shen Kong, Zhi‐Fang Liu, Rong‐Quan Zheng

**Affiliations:** ^1^ Key Lab of Wildlife Biotechnology and Conservation and Utilization of Zhejiang Province Jinhua Zhejiang China; ^2^ Institute of Ecology Zhejiang Normal University Jinhua Zhejiang China; ^3^ Xingzhi College of Zhejiang Normal University Jinhua Zhejiang China

**Keywords:** introgressive hybridization, mitochondrial DNA, nuclear DNA, *Quasipaa*

## Abstract

Introgression may lead to discordant patterns of variation among loci and traits. For example, previous phylogeographic studies on the genus *Quasipaa* detected signs of genetic introgression from genetically and morphologically divergent *Quasipaa shini* or *Quasipaa spinosa*. In this study, we used mitochondrial and nuclear DNA sequence data to verify the widespread introgressive hybridization in the closely related species of the genus *Quasipaa*, evaluate the level of genetic diversity, and reveal the formation mechanism of introgressive hybridization. In Longsheng, Guangxi Province, signs of asymmetrical nuclear introgression were detected between *Quasipaa boulengeri* and *Q. shini*. Unidirectional mitochondrial introgression was revealed from *Q. spinosa* to *Q. shini*. By contrast, bidirectional mitochondrial gene introgression was detected between *Q. spinosa* and *Q. shini* in Lushan, Jiangxi Province. Our study also detected ancient hybridizations between a female *Q. spinosa* and a male *Q. jiulongensis* in Zhejiang Province. Analyses on mitochondrial and nuclear genes verified three candidate cryptic species in *Q. spinosa*, and a cryptic species may also exist in *Q. boulengeri*. However, no evidence of introgressive hybridization was found between *Q. spinosa* and *Q. boulengeri*. *Quasipaa exilispinosa* from all the sampling localities appeared to be deeply divergent from other communities. Our results suggest widespread introgressive hybridization in closely related species of *Quasipaa* and provide a fundamental basis for illumination of the forming mechanism of introgressive hybridization, classification of species, and biodiversity assessment in *Quasipaa*.

## 1 INTRODUCTION

Introgressive hybridization was first described by Anderson and Hubricht (1938) in a study on plant hybridization. Since then, introgressive hybridization has been widely detected in amphibians, birds, and mammals (e.g., Dowling et al., 2016; Matosiuk, Sheremetyeva, Sheremetyev, Saveljev, & Borkowska, 2014; Mccracken, Wilson, & Martin, 2013; Sequeira et al., 2011; Marshall et al., 2011). In the initial study of introgressive hybridization, Mecham (1960) used the morphological analysis of the green tree frog *Hyla cinerea* and the barking tree frog *Hyla gratiosa*; the research has revealed the strong signal of introgressive hybridization between the two species. The rise of genomics age (Walsh, 2001) has aided in understanding introgressive hybridization in multiple evolutionary tiers (Cui et al., 2013) and between diverging species (Magalhaes, Ornelas‐Garcıa, Leal‐Cardin, Ramírez, & Barluenga, 2015). Fitzpatrick and Shaffer (2007) revealed the hybridization between native California tiger salamanders (*Ambystoma californiense*) and introduced barred tiger salamanders (*Ambystoma mavortium*); the hybrid offspring has higher survival rates than most native or introduced individuals. Interspecific hybridization is a force that erodes biodiversity (Dowling et al., 2016). However, many authors regard introgressive hybridization as a creative factor by introducing an unprecedented genetic material during speciation and exploring mechanisms of gene flow between populations (Arnold, 1997; Jiggins & Mallet, 2000; Templeton, 1989). This perspective is particularly common in botanical studies, and botanists verify that groups of interbreeding species of ecological, morphological, genetic, and evolutionary differences are not influenced by extensive hybridization (Hedrick, 2013; Templeton, 1989).

Introgressive hybridization confuses our capability in verifying accurate phylogenetic relationships (Smith, 1992) and leads to mitochondrial DNA (mtDNA) conflicts with nuclear DNA data or vice versa (Chen, Bi, & Fu, 2009; Chen, Ke, & Jinzhong, 2009). Neutral markers can flow from one species to another. Since the emergence of phylogeography as a discipline, mtDNA has been considered an evolutionarily nearly neutral marker that is used mainly to infer evolutionary and demographic history of populations and species (Avise, 2004). Introgressive hybridization has been discovered in many types of organisms on the basis of mtDNA data (Scheffer & Lewis, 2001; Murray et al., 2008; Witt & Hebert, 2011). Inheritance patterns of mtDNA frequently introgress more rapidly than paternally inherited components of the nuclear genome (Bryson, de Oca, Jaeger, & Riddle, 2010), and mtDNA introgression also occurs from one species to another without revealing any trace of nuclear introgression (Ballard & Whitlock, 2004; Chen, Bi, et al., 2009; Chen, Ke, et al., 2009; Liu et al., 2010). However, mtDNA also has its disadvantages. If the species contain mitochondrial pseudogenes, these pseudogenes may amplify or even replace the authentic target mtDNA (Hlaing et al., 2009). Regardless, if a gene or the entire mitochondrial genome is used, the hereditary information of mtDNA only comes from the matriarchal parent and thus has great randomness in studies on population evolution (Granero‐Porati & Porati, 1988). To compensate for the shortcomings of mtDNA, easy‐to‐design primers and high polymorphism in nuclear DNA sequences were used for detecting introgressive hybridization. However, nuclear genes compared with mtDNA are not prone to infiltrate in the process of biological evolution. Therefore, the coevolution between the mitochondrial and nuclear genomes is often disrupted by introgression (Liu et al., 2010). For nearly a decade, mtDNA in combination with nuclear genes exhibited signs of introgressive hybridization among species in which gene flow was unknown (Che et al., 2009, 2010).

The *Quasipaa* is a genus of frogs in the Dicroglossidae family. The genus has no established common name, but many individual species are referred to as spiny frogs. They consist of 11 species distributed in east and southeast Asia, from Thailand and Cambodia to southern and eastern China (Frost, 2014; Hu, Dong, Kong, Mao, & Zheng, 2017; Kong, Zheng, & Zhang, 2016). Most species of *Quasipaa* inhabit rocky streams and mountains from 300 to 1,900 m above sea level (Fei, 2012), and they are likely to possess high genetic divergences between populations (Che et al., 2009). These frogs also have highly nutritional and medicinal values recorded in the compendium of Materia Medica (Li, 2006). In this paper, we studied five species in a widespread overlapping area in southern China. *Quasipaa spinosa* (David, 1875) is mainly distributed in the east of the Yunnan–Kweichow Plateau and the south of the Yangtze River. *Quasipaa boulengeri* (Günther 1889) is mainly located in the middle of South China. These two species share numerous secondary contact regions. *Quasipaa shini* (Ahl, 1930) is mainly spread over two contact zones between the south of Guizhou or Hunan Province and the north of Guangxi Province. *Quasipaa jiulongensis* is mainly found in the southwest of Zhejiang Province and the northern border of Jiangxi and Fujian provinces. *Quasipaa exilispinosa* mainly inhabits a narrow area from northwest to southeast of Fujian Province, Hunan (Yizhang), Guangxi (Longsheng), Guangdong (Ruyuan, Longmen), and Hong Kong. These species share similar morphology. In the last decade, numerous scholars have investigated morphological taxonomy (Huang, 2012), reproduction (Yu et al., 2008), and molecular phylogenetics (Che et al., 2010; Ye et al., 2013; Yu, Zheng, Zhang, Shen, & Dong, 2014; Yu et al., 2016); however, the direction and the level of gene flow between species of *Quasipaa* have yet to be determined.

In our preliminary study, results indicated that one sample of *Q. spinosa* in the contact zone exhibits the mtDNA genotype of *Q. shini*, and one *Q. shini* individual presents the mtDNA genotype of *Q. spinosa* (Ye et al., 2013). Therefore, we hypothesized extensive introgressive hybridization in the species of *Quasipaa*. To verify our hypothesis, we captured a large sample of specimens that were distributed from west to east of South China and studied them using mtDNA and nuclear genes to test whether introgressive hybridization extensively occurs in *Quasipaa* in the sympatric distribution region. We also investigated the distribution region and survival environment of the genus *Quasipaa* to obtain further information on genetic diversity. This study provides a fundamental basis for elucidating the forming mechanism of introgressive hybridization, classification of species, and genetic diversity assessment in *Quasipaa*.

## 2 MATERIALS AND METHODS

### 2.1 Sampling of specimens

**Figure 1:**
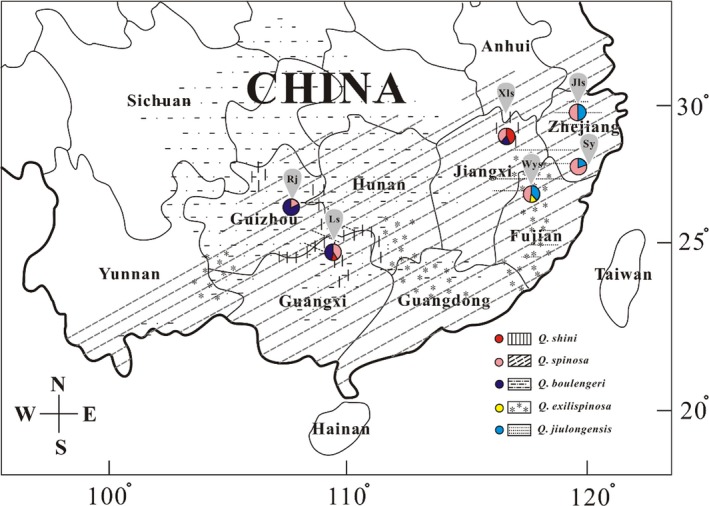
Map of sampling based on morphology localities for this study. The background represents the distribution of five species of *Quasipaa*. The pie chart shows the proportion and category of the sample. Six localities are grouped by city, with the following codes: Jls, Jiulongshan; Sy, Songyang; Wys, Wuyishan; Xls, Lushan; Ls, Longsheng; and Rj, Rongjiang

A total of 299 individuals of the genus *Quasipaa* were obtained from six locations across South China from 2013 to 2015 (Table 1 and Figure 1). Fieldwork was mainly conducted in the first 5–10 months, rendering the collection of frogs in breeding condition. All frogs were initially identified by morphological traits and then labeled by haplotype numbers. Dead frogs were frozen at −80°C, and surviving frogs were fed for subsequent DNA sequencing and hybridization. Four species from *Fejervarya limnocharis*,* Hoplobatrachus rugulosus*,* Nanorana parkeri*, and *Nanorana quadranus* were chosen as outgroup taxa based on Frost et al. (2006). We obtained the outgroup sequences from GenBank (Appendix S1).

**Table 1:** GPS coordinates and sample size for six sampling sites in southern China

Location	Coordinates		*Q. spinosa*	*Q. exilispinosa*	*Q. boulengeri*	*Q. jiulongensis*	*Q. shini*
Jiulongshan	E118°53′21″,	N28°21′41″	25	0	0	23	0
Songyang	E119°29′7″,	N28°27′23″	36	0	0	9	0
Wuyishan	E118°0′36″,	N27°16′12″	28	8	0	21	0
Lushan	E116°13′19″,	N29°40′06″	18	0	9	0	24
Longsheng	E109°58′48″,	N25°49′12″	30	0	40	0	8
Rongjiang	E108°30′54″,	N25°56′17″	6	0	23	0	0
Total			143	8	72	44	32

### 2.2 DNA extraction, PCR, and sequencing

Genomic DNA was extracted from the muscles of dead frogs by standard three‐step phenol–chloroform extraction (Sambrook & Russell, 2001) or noninvasive sampling of individual survival using Genomic DNA kits (Sangon) in accordance with the manufacturer's protocol. The parameters of DNA were assessed via a DNA spectrophotometer, and DNA was stored at −20°C. We amplified and sequenced two fragments of mtDNA (partial sequences of 12S and 16S ribosomal RNA genes) and two nuclear DNAs (partial sequence of recombinase activating 1 protein gene and a single exon 1 of rhodopsin gene).

Polymerase chain reaction amplification of mtDNA fragments was conducted in a 25‐ml volume reaction under the following conditions: initial denaturation for 4 min at 95°C, 35 cycles of denaturation for 30 s at 94°C, annealing for 30 s at 58°C, extension for 1 min at 72°C, and a final extension for 10 min at 72°C. Amplification of nuclear genes was performed using the same PCR protocols except for the denaturation for 1 min at 94°C and annealing for 1 min at 58°C (*Rag‐1*) and 60°C (*Rhodopsin*). The primer sequences of mtDNA and nuclear genes are provided in Table 2. PCR products were visualized with a 1% agarose gel, and products were purified using an Axygen DNA kit (Invitrogen, Shanghai, China). We sequenced amplified fragments with PCR primers using a commercial sequencing service in both directions (Sangon Biotech, Shanghai, China) and then determined the identity of raw sequences to BLAST searches in GenBank.

**Table 2:** Primers used for mitochondrial and nuclear DNA amplification in this study

Locus	Primer	primer sequences (5′‐3′)	Size (bp)	Source
*16S rRNA*	Zh4823	5′‐CGCCTGTTTACCAAAAACAT‐3′5′‐CTCCGGTCTGAACTCAGATC‐3′	557	Bossuyt and Milinkovitch (2000)
*12SrRNA*	Ch4823	5′‐AGTGCTGAAAACGCTAAGAC‐3′5′‐AGGGCGACGGGCGGTGTGTAC‐3′	841	Zhou, Zhang, Zheng, Yu, and Yang (2009), Kocher et al.(1989)
*Rag‐1*	Amp	5′‐ACAGGATATGATGARAAGCTTGT‐3′5′‐TCGCGTTCGATGATCTCTGG‐3′	730	Hoegg, Vences, Brinkmann, and Meyer (2004)
*Rhodopsin*	Rhod	5′‐ACCATGAACGGAACAGAAGGYCC‐3′5′‐GTAGCGAAGAARCCTTCAAMGTA‐3′	318	Bossuyt and Milinkovitch (2000)

### 2.3 Sequence alignment and phylogenetic analyses

Amplified fragments of mitochondrial and nuclear genes were aligned using ClustalX1.81 (Thompson, Gibson, Plewniak, Jeanmougin, & Higgins, 1997) and then aligned manually using MEGA 6.0 (Tamura, Stecher, Peterson, Filipski, & Kumar, 2013). Nucleotide and haplotype diversities of each taxon and population were calculated using DnaSP 5.0 (Librado & Rozas, 2009). We conducted phylogenetic relationships among haplotypes using maximum parsimony (MP), maximum likelihood (ML), and Bayesian inference (BI). Heuristic MP searches with tree bisection and reconnection branch swapping in PAUP*4.0 (Swofford, 2002) were executed. The stability of support values was estimated from nonparametric bootstrap replications using PAUP*4.0 with 100 replicates. ML analyses were performed using PAUP*4.0 (Swofford, 2002) with the best‐fitting model (mtDNA: GTR+I+G, nuclear DNA: HKY+I+G) by the Akaike information criterion (AIC) in Modeltest 3.7 (Posada & Crandall, 1998). To display information on the relationships between markers with genetic diversity, a nuclear network was created with neighbor‐net (Huson & Bryant, 2006) using SplitsTree (ver. 4.1). The best nucleotide substitution model of AIC was to be used. Bayesian analysis was performed in MrBayes3.1.2 with the best‐fitting model (mtDNA: GTR+I+G, nuclear DNA: K80) using four incrementally heated Markov Chain Monte Carlo for 6,000,000 generations with sampling trees every 100th generation. The first 50% were discarded as burn‐in, and the Bayesian posterior probabilities were estimated by the remaining trees.

## 3 RESULTS

### 3.1 Sequence characteristics

All 299 specimens were sequenced for the mtDNA (i.e., *12srRNA* and *16srRNA*) and nuclear DNA (i.e., *Rag‐1* and *Rhodopsin*) fragments. Sequences of the *12srRNA* fragment were 841 bp in length, and the ambiguous fragments of 73 bp were excluded from further analysis. The 557 bp of *16srRNA* was produced, and the ambiguous alignment of one region with 24 bp was excluded from further analysis. The total amplified mtDNA includes 370 variable sites and 82 parsimony informative sites. All 299 specimens of two nuclear fragments were successfully sequenced. A total of 730 bp of *Rag‐1* and 315 bp of *rhodopsin* were used for analyses, and 109 bp of *Rag‐1* and 3 bp of *Rhodopsin* were excluded from further analysis due to ambiguous alignment. The total alignment consisted of two nuclear fragments for the dataset in which 195 sites were variable and 119 sites were potentially phylogenetically informative.

### 3.2 Genetic distances

Genetic distances of *Quasipaa* with mitochondrial and nuclear DNA were calculated following the Tamura and Nei (1993) distance module in MEGA 6.0 (Tamura et al., 2013). The average genetic distance between all mitochondrial sequences ranged from 2.4% to 7.5% (Table 3), and the intraspecific genetic distance of *Q. spinosa* (3.6%) and *Q. shini* (3.2%) was greater than the smallest genetic distance of the species (2.4%). The average genetic distance of nuclear sequences ranged from 0.9% to 3.0%. Interestingly, the mean genetic distance between *Q. shini* and *Q. boulengeri* was 0.9%; however, the intraspecific genetic distance of *Q. shini* was 0.9% and that of *Q. boulengeri* was 1.1%.

**Table 3:** Population parameters of divergence for genus *Quasipaa* based on the fragment of mitochondrial/nuclear gene (median, %)

Species	*Q. spinosa*	*Q. jiulongensis*	*Q. boulengeri*	*Q. shini*	*Q. exilispinosa*
*Q. spinosa*	3.6/1.1				
*Q. jiulongensis*	2.9/1.1	2.4/0.8			
*Q. boulengeri*	5.5/1.7	7.2/2.3	2.0/1.1		
*Q. shini*	3.5/1.8	6.0/1.8	5.5/0.9	3.2/0.9	
*Q. exilispinosa*	2.4/0.9	5.2/1.5	7.5/3.0	6.3/3.0	1.1/0.3

### 3.3 Phylogenetic relationships and network

**Figure 2:**
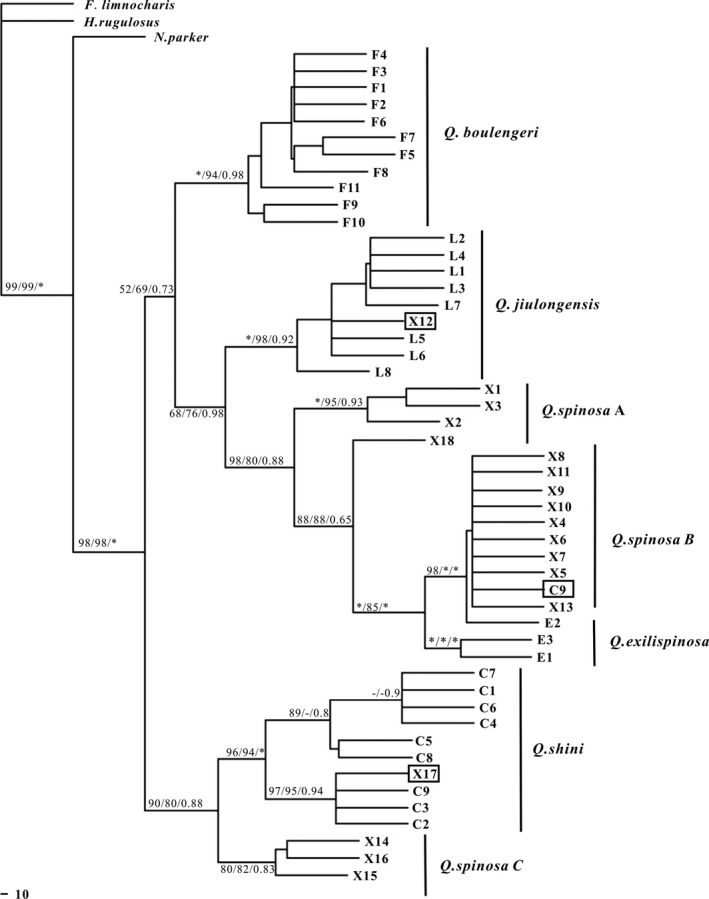
Phylogenetic relationships of haplotypes in genus *Quasipaa* based on the mitochondrial DNA sequences, as determined by MP, ML, and Bayesian inference. Numbers indicate the percentage confidence level of each node estimated by 1,000 bootstrap samplings of the data. Bootstrap support values over 50% were provided. Legend: asterisk (*) indicates 100% ML and MP bootstrap support and 1.0 Bayesian posterior probabilities. “F,” haplotypes of *Q. boulengeri*; “X,” haplotypes of *Q. spinosa*; “L,” haplotypes of *Q. jiulongensis*; “E,” haplotypes of *Q. exilispinosa*; and “C,” haplotypes of *Q. shini*

**Figure 3:**
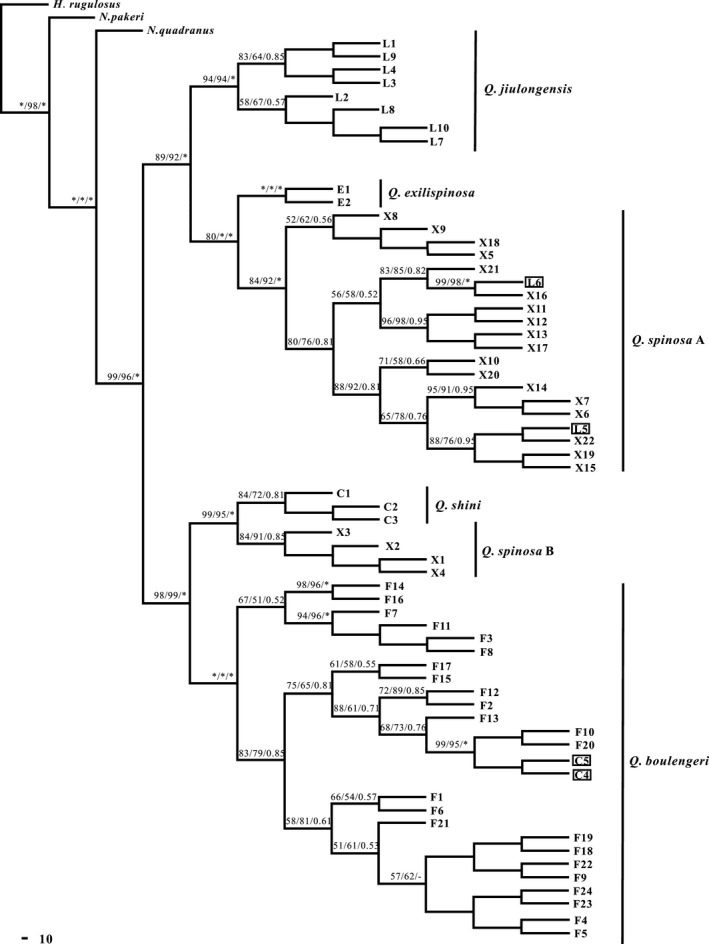
Phylogenetic relationships of haplotypes in genus *Quasipaa* based on the DNA sequences, as determined by MP, ML, and Bayesian inference. Numbers indicate the percentage confidence level of each node estimated by 1,000 bootstrap samplings of the data. Bootstrap support values over 50% were provided. Legend: asterisk (*) indicates 100% ML and MP bootstrap support and 1.0 Bayesian posterior probabilities. “F,” haplotypes of *Q. boulengeri*; “X,” haplotypes of *Q. spinosa*; “L,” haplotypes of *Q. jiulongensis*; “E,” haplotypes of *Q. exilispinosa*; and “C,” haplotypes of *Q. shini*

Based on the haplotype data, we analyzed every gene of MP, BI, and ML, and the results of the analysis generated almost identical tree topology (Figures 2 and 3). MtDNA consisted of seven major haplotype clades, whereas nuclear DNA consisted of six major haplotype clades. In the mtDNA topology, *Q. spinosa* A included all haplotypes of *Q. spinosa* from Zhejiang, Jiangxi, and Guizhou provinces. *Q. spinosa* B included *Q. spinosa* from Zhejiang, Fujian, Guangxi, and Jiangxi provinces, except one haplotype of *Q. shini* C9. *Q. spinosa* C included *Q. spinosa* from Guizhou and Guangxi provinces. Results supported the presence of three candidate cryptic species of *Q. spinosa* (Ye et al., 2013), each of which corresponded to a geographical region. The clade of *Q. jiulongensis* included all haplotypes of *Q. jiulongensis* from Zhejiang Province, except *Q. spinosa* X12. The clade of *Q. exilispinosa* included all haplotypes of *Q. exilispinosa* from Fujian, whereas the clade of *Q. shini* included all haplotypes of *Q. shini* from Guangxi and Jiangxi, except *Q. spinosa* X17. The clade of *Q. boulengeri* included all haplotypes of *Q. boulengeri* from Guangxi, Jiangxi, and Guizhou.

**Figure 4:**
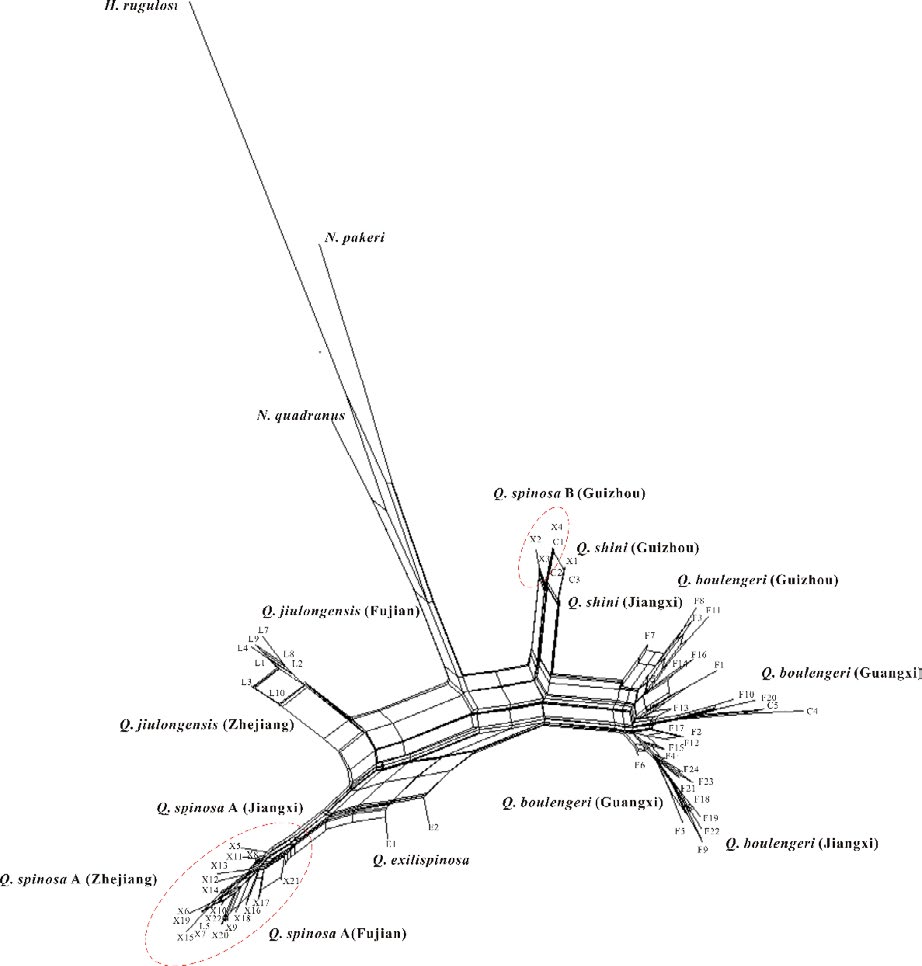
Nuclear DNA neighbor‐net network created using maximum likelihood distances. Oval highlights clade that contains two *spinosa* species

In the phylogenetic relationships of the nuclear DNA, *Q. spinosa* consisted of *Q. spinosa* A (Zhejiang, Fujian, and Jiangxi provinces) and *Q. spinosa* B (Guangxi, Guizhou provinces). The nuclear network is similar to the nuclear gene tree (Figure 4) and strongly supports the existence of cryptic lineages in *Q. spinosa*. The clade of *Q boulengeri* included all haplotypes of *Q. boulengeri* from Guangxi, Guizhou, and Jiangxi, except two haplotypes of *Q. shini* C4 and C5 (Figures 3 and 4). All samples of *Q. exilispinosa* (Fujian), *Q. jiulongensis* (Zhejiang and Fujian), and *Q. shini* (Guangxi and Jiangxi) were classified into three separate haplotype clades, except for *Q. jiulongensis* L5 and L6, which were distributed to *Q. spinosa*.

## 4 DISCUSSION

Introgressive hybridization has been detected in a variety of isolated populations that have come into secondary contact (Rubidge & Taylor, 2004) and has been regarded as a creative factor by introducing a new genetic material during speciation (Arnold, 1997; Jiggins & Mallet, 2000; Templeton, 1989). At present, molecular markers offer an opportunity to reveal gene flow in divergent lineages and infer introgressive hybridization by detecting topological incongruence between gene trees (Choleva et al., 2014). The incongruence may be caused by the overall amount of differentiation (Toews & Brelsford, 2012). In the present study, we analyzed the phylogenetic relationships of mtDNA. The haplotype sharing between *Q spinosa* and *Q. shini* has been detected in analysis of mtDNA dates (Figure 2), and our results were consistent with the *12srRNA* and *16srRNA* sequences in the study of Ye et al. (2013). By contrast, the morphological characteristics of *Q. spinosa* or *Q. shini* had not detected the gene flow in nuclear gene. The network of nuclear is presented in a radial shape (Figure 3), indicating that there are obvious expansions of each species. The population parameters of divergence for genus *Quasipaa* based on the fragment of mitochondrial and nuclear gene have showed that intraspecific genetic distance of *Q. spinosa* was greater than interspecific genetic distance between *Q. spinosa* and *Q. shini* (Table. 3). We infer that the differentiation of genetic lineage in *Quasipaa* is in contact with each other at the later stage of differentiation, leading to hybridization and gene introgression, resulting in incongruence between mitochondrial and nuclear gene. Our results do not exclude ancient introgression between *Q. spinosa* (or *Q. shini*) and previously sympatric species. The gradients of population density, asymmetry of hybridization, or differential adaptation of the parents between the parental taxa may cause hybrid zone movement (Barton & Hewitt, 1985; Key, 1968), but we did not specifically investigate the size of the habitat and population density. So related field work in the further will help to reveal the specific mechanism. However, the anomalous results for *Q. spinosa* and *Q. shini* can also be explained by incomplete lineage sorting. If the interspecific segregation is complete, even then, both species retain more common ancestor polymorphism, the differentiation of species is less than that of interspecific populations (Muir & Schlötterer, 2005).

Differences in morphological characteristics between *Q. spinosa* and *Q. jiulongensis* are mainly concentrated in the roughness of back skin and individual size. The roughness of back skin and individual size of *Q. spinosa* is evident, and the back of *Q. jiulongensis* has four or five tan markings that are symmetrically arranged (Fei, 2012). In the present study, the haplotype sharing between *Q spinosa* and *Q. jiulongensis* has been detected in analysis of DNA dates (Figures 2 and 3). Although young species complexes between *Q. spinosa* and *Q. jiulongensis* have not been found in the wild or through artificial breeding, a conflict between morphological and molecular data in *Q. spinosa* and *Q. jiulongensis* has been detected. We inferred that nucleocytoplasmic inconsistencies or ancient introgression had occurred in a male *Q. spinosa* and a female *Q. jiulongensis* in historical evolution and that intraspecific mating in hybrid offspring produces no viable offspring while hybrid offspring mating with a male *Q. spinosa* produces fully fertile hybrid offspring. Female preference matches the unusual system with female hybrid offspring preferring to mate with a male *Q. spinosa*. This response may be beneficial because it allows the female hybrid offspring to increase their own residual reproductive value through a second mating with a male *Q. spinosa* in the same breeding season. The example has occurred in the water frog complex between *Rana lessonae* and *Rana ridibunda*. The hybrid offspring may be more prone to mate with a male *R. lessonae* (Reyer, Frei, & Som, 1999). Recent studies have also demonstrated that the mitochondrial genome of *Stenella clymene* is more closely related to that of *Stenella coeruleoalba*, whereas the nuclear genome is more closely related to *Stenella longirostris* (Amaral, Gretchen, Coelho, George, & Rosenbaum, 2014). This phenomenon may be interpreted as an ancient hybridization between a female *Stenella coeruleoalba* and a male *S. longirostris*. Discrepancies were also found in the hybrid origin of *Trachemys* and *Hyla arborea* (Gvoždík et al., 2015; Parham et al., 2013).

The morphological characteristics of *Q. boulengeri* were similar to those of *Q. shini*, and the most obvious difference was the location of the secondary sexual characteristics of the keratinized spines on the toe (Fei, 2012). The haplotype of *Q. shini* was detected in that of *Q. boulengeri* on the basis of the present study's nuclear genetic analysis (Figure 3); however, the opposite situation of mitochondrial DNA was not detected. Thus, we inferred that the unidirectional introgression of nuclear genes infiltrated from *Q. boulengeri* to *Q. shini*. The directional introgression between *Q. boulengeri* and *Q. shini* may be due to the following two reasons: (I) the degree of gene flow from *Q. boulengeri* was greater than that of *Q. shini* to *Q. boulengeri*. For a long time, gene flow from *Q. shini* to *Q. boulengeri* was hidden by that from *Q. boulengeri* to *Q. shini*. The dominant manner in gene introgression of *Q. boulengeri* may play a leading role in hybridization; dominant species have a greater capacity to adapt to environmental changes than other species, and genes that encode dominant species can easily pass to other species (Salvini et al., 2009). The hybrid offspring of *Q. boulengeri* and *Q. shini* would rather mate with *Q. boulengeri* than with *Q. shini*. This biased gene introgression was also detected in *Abramis bram* (Demandt & Bergek, 2009). (II) This phenomenon is an evidence of a biased diffusion of *Q. boulengeri* (biased diffusion refers to a speculation that the male diffusion of *Q. boulengeri* is the major mechanism in the sympatric distribution zone between *Q. boulengeri* and *Q. shini* or part of the female *Q. boulengeri* is also involved in the diffusion process). Population expansion of *Q. boulengeri* and *Q. shini* possibly entered the sympatric distribution zone. Given that backcross progenies constantly passed the nuclear genes of *Q. boulengeri* to the gene pool of *Q. shini*, populations of *Q. shini* were relatively local, which may cause the nuclear genes of *Q. shini* to be similar to those of *Q. boulengeri*. Research has revealed that genes of a relatively small population may be diluted or replaced by dominant species during genetic assimilation in the introgressive hybridization of two species, thereby reducing biodiversity (Wang & Peng, 2003). The adverse effects of one‐way introgressive hybridization still need to be clarified further in future research.

Morphological characteristics are factors of species identification (Rubidge & Taylor, 2004). Different geographical populations have adapted to their living environments, thereby altering intraspecific genetic composition. In the phylogenetic analysis of mtDNA, *Q. spinosa* may also divide into three independent clades based on the corresponding geography, which consists with the research of Ye et al. In addition, the tree topology of the nuclear gene showed that the population of *Q. boulengeri* was divided into three branches; this result also agrees with the suggestion of four major matrilines of *Q. boulengeri* (Yan et al., 2012). The incomplete lineage sorting and the ancestral polymorphism were to be considered in *Quasipaa*; sex‐biased dispersal, human introductions, hybrid offspring infertility, and low viability or low fertility also resulted in the directional or nondirectional gene introgression pattern in *Quasipaa* (Toews & Brelsford, 2012). In the present study, introgression between *Q. exilispinosa* and other species of *Quasipaa* (e.g., *Q. jiulongensis* and *Q. spinosa*) was not detected. This phenomenon may be due to two reasons. First, the samples of *Q. exilispinosa* were inadequate. Second, the morphological divergence between *Q. exilispinosa* and other species of *Quasipaa* was evident, which may hinder mating between *Q. exilispinosa* and other syntrophic species. The specific reasons need further studies.

Introgressive hybridization is a manner of speciation that reveals fitness of hybrid offspring, analyzes genetic differences between hybrid offspring and pure offspring via microsatellite or genome scanning, and tests hybrid offspring as a new species. These questions need to be researched and solved urgently. Given the special physical and life history characteristics of amphibious animals, their sensitivity to the external environment is significantly higher than that in other species (Schmidt, 2000). In China, the main leading threats to amphibians are habitat degeneration and loss, human capture, and pollution. Protection zones such as Mount Wuyi (Fujian Province) and Mount Jiulong (Zhejiang Province), which have relatively high biodiversity, should be protected and prioritized.

## CONFLICT OF INTEREST

None declared.

## AUTHOR CONTRIBUTION

R. Q. Zheng and W. F. Hu conceived and designed the experiments. Q. P. Zhang, T. T. Zhou, and Z. F. Liu performed the experiments. R. Q. Zheng, Q. P. Zhang, T. T. Zhou, and S. S. Kong analyzed the data and wrote the manuscript. All authors read and approved the final manuscript.
