# Supplementary material for: Interspecies introgressive hybridization in spiny frogs Quasipaa (Family Dicroglossidae) revealed by analyses on multiple mitochondrial and nuclear genes
Source: Ecol Evol. 2017 Dec 21;8(2):1260–70. doi: 10.1002/ece3.3728 (PMC5773314; doi:10.1002/ece3.3728)
Supplement: Supplementary file 1 [file ECE3-8-1260-s001.docx]

Appendix 1 Sampling information in *Quasipaa* including specimen ID, localities, haplotypes or GenBank accession numbers.

| Specimen  ID | Locality | Coordinates | nuclear genes | Mitochondrial genes |
| --- | --- | --- | --- | --- |
| Outgroups |  |  |  |  |
| *Fejervarya limnocharis* | No data | No data |  | AB277302.1 AB277286.1 |
| *Hoplobatrachusrugulosus* | No data | No data | KJ637297.1 EU979933.1 | AB636618.1 AB636609.1 |
| *Nanorana parkeri* | No data | No data | DQ019501.1 EU979872.1 | NC026789.1 NC026789.1 |
| *Nanorana quadranus* | No data | No data | HM163591.1 EU979890.1 |  |
| Ingroups |  |  |  |  |
| Genus *Quasipaa* |  |  |  |  |
| *Q.exilispinosa* |  |  |  |  |
| xj1 | Wuyishan | E118°0′36″, N27°16′12″ | E1 | E3 |
| xj2 | Wuyishan | E118°0′36″, N27°16′12″ | E1 | E3 |
| xj3 | Wuyishan | E118°0′36″, N27°16′12″ | E2 | E1 |
| xj4 | Wuyishan | E118°0′36″, N27°16′12″ | E1 | E2 |
| xj5 | Wuyishan | E118°0′36″, N27°16′12″ | E1 | E1 |
| xj6 | Wuyishan | E118°0′36″, N27°16′12″ | E2 | E1 |
| xj7 | Wuyishan | E118°0′36″, N27°16′12″ | E2 | E1 |
| xj8 | Wuyishan | E118°0′36″, N27°16′12″ | E2 | E3 |
| Q.spinosa |  |  |  |  |
| GX1 | Rongjiang | E108°30′54″,  N 25°56′17″ | X1 | X14 |
| GX2 | Rongjiang | E108°30′54″,  N 25°56′17″ | X2 | X14 |
| GX3 | Rongjiang | E108°30′54″,  N 25°56′17″ | X2 | X15 |
| GX4 | Rongjiang | E108°30′54″,  N 25°56′17″ | X1 | X16 |
| GX5 | Rongjiang | E108°30′54″,  N 25°56′17″ | X3 | X15 |
| GX6 | Rongjiang | E108°30′54″,  N 25°56′17″ | X3 | X15 |
| LX1 | Lushan | E116°13′19″, N29°40′06″ | X5 | X5 |
| LX2 | Lushan | E116°13′19″, N29°40′06″ | X5 | X8 |
| LX3 | Lushan | E116°13′19″, N29°40′06″ | X8 | X8 |
| LX4 | Lushan | E116°13′19″, N29°40′06″ | X6 | X12 |
| LX5 | Lushan | E116°13′19″, N29°40′06″ | X9 | X12 |
| LX6 | Lushan | E116°13′19″, N29°40′06″ | X9 | X1 |
| LX7 | Lushan | E116°13′19″, N29°40′06″ | X9 | X1 |
| LX8 | Lushan | E116°13′19″, N29°40′06″ | X6 | X1 |
| LX9 | Lushan | E116°13′19″, N29°40′06″ | X8 | X12 |
| LX10 | Lushan | E116°13′19″, N29°40′06″ | X10 | X2 |
| LX11 | Lushan | E116°13′19″, N29°40′06″ | X11 | X12 |
| LX12 | Lushan | E116°13′19″, N29°40′06″ | X9 | X2 |
| LX13 | Lushan | E116°13′19″, N29°40′06″ | X9 | X8 |
| LX14 | Lushan | E116°13′19″, N29°40′06″ | X6 | X8 |
| LX15 | Lushan | E116°13′19″, N29°40′06″ | X8 | X15 |
| LX16 | Lushan | E116°13′19″, N29°40′06″ | X9 | X14 |
| LX17 | Lushan | E116°13′19″, N29°40′06″ | X9 | X12 |
| LX18 | Lushan | E116°13′19″, N29°40′06″ | X6 | X8 |
| gx1 | Longsheng | E109°58′48″, N25°49′12″ | X3 | X5 |
| gx2 | Longsheng | E109°58′48″, N25°49′12″ | X1 | X6 |
| gx3 | Longsheng | E109°58′48″, N25°49′12″ | X3 | X6 |
| gx4 | Longsheng | E109°58′48″, N25°49′12″ | X3 | X6 |
| gx5 | Longsheng | E109°58′48″, N25°49′12″ | X7 | X6 |
| gx6 | Longsheng | E109°58′48″, N25°49′12″ | X3 | X6 |
| gx7 | Longsheng | E109°58′48″, N25°49′12″ | X4 | X14 |
| gx8 | Longsheng | E109°58′48″, N25°49′12″ | X7 | X14 |
| gx9 | Longsheng | E109°58′48″, N25°49′12″ | X7 | X6 |
| gx10 | Longsheng | E109°58′48″, N25°49′12″ | X7 | X6 |
| gx11 | Longsheng | E109°58′48″, N25°49′12″ | X3 | X6 |
| gx12 | Longsheng | E109°58′48″, N25°49′12″ | X4 | X6 |
| gx13 | Longsheng | E109°58′48″, N25°49′12″ | X4 | X15 |
| gx14 | Longsheng | E109°58′48″, N25°49′12″ | X7 | X15 |
| gx15 | Longsheng | E109°58′48″, N25°49′12″ | X7 | X6 |
| gx16 | Longsheng | E109°58′48″, N25°49′12″ | X7 | X6 |
| gx17 | Longsheng | E109°58′48″, N25°49′12″ | X3 | X6 |
| gx18 | Longsheng | E109°58′48″, N25°49′12″ | X4 | X6 |
| gx19 | Longsheng | E109°58′48″, N25°49′12″ | X3 | X6 |
| gx20 | Longsheng | E109°58′48″, N25°49′12″ | X3 | X6 |
| gx21 | Longsheng | E109°58′48″, N25°49′12″ | X3 | X6 |
| gx22 | Longsheng | E109°58′48″, N25°49′12″ | X3 | X6 |
| gx23 | Longsheng | E109°58′48″, N25°49′12″ | X3 | X6 |
| gx24 | Longsheng | E109°58′48″, N25°49′12″ | X7 | X6 |
| gx25 | Longsheng | E109°58′48″, N25°49′12″ | X3 | X6 |
| gx26 | Longsheng | E109°58′48″, N25°49′12″ | X7 | X16 |
| gx27 | Longsheng | E109°58′48″, N25°49′12″ | X7 | X16 |
| gx28 | Longsheng | E109°58′48″, N25°49′12″ | X4 | X16 |
| gx29 | Longsheng | E109°58′48″, N25°49′12″ | X4 | X5 |
| gx30 | Longsheng | E109°58′48″, N25°49′12″ | X7 | X5 |
| Jx1 | Jiulongshan | E118°53′21″, N28°21′41″ | X10 | X10 |
| Jx2 | Jiulongshan | E118°53′21″, N28°21′41″ | X13 | X4 |
| Jx3 | Jiulongshan | E118°53′21″, N28°21′41″ | X10 | X4 |
| Jx4 | Jiulongshan | E118°53′21″, N28°21′41″ | X12 | X2 |
| Jx5 | Jiulongshan | E118°53′21″, N28°21′41″ | X13 | X2 |
| Jx6 | Jiulongshan | E118°53′21″, N28°21′41″ | X10 | X2 |
| Jx7 | Jiulongshan | E118°53′21″, N28°21′41″ | X10 | X7 |
| Jx8 | Jiulongshan | E118°53′21″, N28°21′41″ | X13 | X7 |
| Jx9 | Jiulongshan | E118°53′21″, N28°21′41″ | X10 | X4 |
| Jx10 | Jiulongshan | E118°53′21″, N28°21′41″ | X21 | X4 |
| Jx11 | Jiulongshan | E118°53′21″, N28°21′41″ | X9 | X4 |
| Jx12 | Jiulongshan | E118°53′21″, N28°21′41″ | X12 | X17 |
| Jx13 | Jiulongshan | E118°53′21″, N28°21′41″ | X9 | X2 |
| Jx14 | Jiulongshan | E118°53′21″, N28°21′41″ | X9 | X2 |
| Jx15 | Jiulongshan | E118°53′21″, N28°21′41″ | X10 | X18 |
| Jx16 | Jiulongshan | E118°53′21″, N28°21′41″ | X12 | X17 |
| Jx17 | Jiulongshan | E118°53′21″, N28°21′41″ | X13 | X12 |
| Jx18 | Jiulongshan | E118°53′21″, N28°21′41″ | X22 | X17 |
| Jx19 | Jiulongshan | E118°53′21″, N28°21′41″ | X12 | X12 |
| Jx20 | Jiulongshan | E118°53′21″, N28°21′41″ | X9 | X3 |
| Jx21 | Jiulongshan | E118°53′21″, N28°21′41″ | X9 | X3 |
| Jx22 | Jiulongshan | E118°53′21″, N28°21′41″ | X10 | X13 |
| Jx23 | Jiulongshan | E118°53′21″, N28°21′41″ | X13 | X13 |
| Jx24 | Jiulongshan | E118°53′21″, N28°21′41″ | X10 | X11 |
| Jx25 | Jiulongshan | E118°53′21″, N28°21′41″ | X12 | X4 |
| Jx26 | Jiulongshan | E118°53′21″, N28°21′41″ | X13 | X10 |
| Jx27 | Jiulongshan | E118°53′21″, N28°21′41″ | X10 | X4 |
| Sx1 | Songyang | E119°29′7″, N28°27′23″ | X11 | X4 |
| Sx2 | Songyang | E119°29′7″, N28°27′23″ | X12 | X4 |
| Sx4 | Songyang | E119°29′7″, N28°27′23″ | X13 | X4 |
| Sx5 | Songyang | E119°29′7″, N28°27′23″ | X13 | X4 |
| Sx6 | Songyang | E119°29′7″, N28°27′23″ | X13 | X10 |
| Sx7 | Songyang | E119°29′7″, N28°27′23″ | X13 | X4 |
| Sx8 | Songyang | E119°29′7″, N28°27′23″ | X12 | X3 |
| Sx9 | Songyang | E119°29′7″, N28°27′23″ | X14 | X4 |
| Sx10 | Songyang | E119°29′7″, N28°27′23″ | X7 | X4 |
| Sx11 | Songyang | E119°29′7″, N28°27′23″ | X7 | X9 |
| Sx12 | Songyang | E119°29′7″, N28°27′23″ | X11 | X9 |
| Sx13 | Songyang | E119°29′7″, N28°27′23″ | X7 | X3 |
| Sx14 | Songyang | E119°29′7″, N28°27′23″ | X13 | X3 |
| Sx15 | Songyang | E119°29′7″, N28°27′23″ | X13 | X10 |
| Sx16 | Songyang | E119°29′7″, N28°27′23″ | X7 | X4 |
| Sx17 | Songyang | E119°29′7″, N28°27′23″ | X14 | X10 |
| Sx18 | Songyang | E119°29′7″, N28°27′23″ | X9 | X10 |
| Sx19 | Songyang | E119°29′7″, N28°27′23″ | X9 | X13 |
| Sx20 | Songyang | E119°29′7″, N28°27′23″ | X7 | X13 |
| Sx21 | Songyang | E119°29′7″, N28°27′23″ | X13 | X10 |
| Sx22 | Songyang | E119°29′7″, N28°27′23″ | X7 | X9 |
| Sx23 | Songyang | E119°29′7″, N28°27′23″ | X13 | X9 |
| Sx24 | Songyang | E119°29′7″, N28°27′23″ | X9 | X11 |
| Sx25 | Songyang | E119°29′7″, N28°27′23″ | X7 | X13 |
| Sx26 | Songyang | E119°29′7″, N28°27′23″ | X15 | X11 |
| Sx27 | Songyang | E119°29′7″, N28°27′23″ | X16 | X4 |
| Sx28 | Songyang | E119°29′7″, N28°27′23″ | X7 | X13 |
| Sx29 | Songyang | E119°29′7″, N28°27′23″ | X7 | X11 |
| Sx30 | Songyang | E119°29′7″, N28°27′23″ | X13 | X4 |
| Sx31 | Songyang | E119°29′7″, N28°27′23″ | X13 | X13 |
| Sx32 | Songyang | E119°29′7″, N28°27′23″ | X10 | X10 |
| Sx33 | Songyang | E119°29′7″, N28°27′23″ | X10 | X10 |
| Sx34 | Songyang | E119°29′7″, N28°27′23″ | X10 | X13 |
| Sx35 | Songyang | E119°29′7″, N28°27′23″ | X9 | X13 |
| Sx36 | Songyang | E119°29′7″, N28°27′23″ | X9 | X13 |
| WX1 | Wuyishan | E118°0′36″, N27°16′12″ | X9 | X9 |
| WX2 | Wuyishan | E118°0′36″, N27°16′12″ | X9 | X9 |
| WX3 | Wuyishan | E118°0′36″, N27°16′12″ | X17 | X7 |
| WX4 | Wuyishan | E118°0′36″, N27°16′12″ | X8 | X7 |
| WX5 | Wuyishan | E118°0′36″, N27°16′12″ | X9 | X11 |
| WX5 | Wuyishan | E118°0′36″, N27°16′12″ | X9 | X4 |
| WX7 | Wuyishan | E118°0′36″, N27°16′12″ | X13 | X4 |
| WX8 | Wuyishan | E118°0′36″, N27°16′12″ | X13 | X16 |
| WX9 | Wuyishan | E118°0′36″, N27°16′12″ | X13 | X4 |
| WX10 | Wuyishan | E118°0′36″, N27°16′12″ | X18 | X4 |
| WX11 | Wuyishan | E118°0′36″, N27°16′12″ | X17 | X15 |
| WX12 | Wuyishan | E118°0′36″, N27°16′12″ | X8 | X15 |
| WX13 | Wuyishan | E118°0′36″, N27°16′12″ | X19 | X15 |
| WX14 | Wuyishan | E118°0′36″, N27°16′12″ | X10 | X15 |
| WX15 | Wuyishan | E118°0′36″, N27°16′12″ | X18 | X15 |
| WX16 | Wuyishan | E118°0′36″, N27°16′12″ | X17 | X9 |
| WX17 | Wuyishan | E118°0′36″, N27°16′12″ | X9 | X7 |
| WX18 | Wuyishan | E118°0′36″, N27°16′12″ | X17 | X7 |
| WX19 | Wuyishan | E118°0′36″, N27°16′12″ | X16 | X11 |
| WX20 | Wuyishan | E118°0′36″, N27°16′12″ | X16 | X4 |
| WX21 | Wuyishan | E118°0′36″, N27°16′12″ | X20 | X4 |
| WX22 | Wuyishan | E118°0′36″, N27°16′12″ | X9 | X4 |
| WX23 | Wuyishan | E118°0′36″, N27°16′12″ | X17 | X9 |
| WX24 | Wuyishan | E118°0′36″, N27°16′12″ | X8 | X7 |
| WX25 | Wuyishan | E118°0′36″, N27°16′12″ | X18 | X7 |
| WX26 | Wuyishan | E118°0′36″, N27°16′12″ | X16 | X11 |
| WX27 | Wuyishan | E118°0′36″, N27°16′12″ | X9 | X4 |
| WX28 | Wuyishan | E118°0′36″, N27°16′12″ | X16 | X4 |
| WX29 | Wuyishan | E118°0′36″, N27°16′12″ | X9 | X4 |
| WX30 | Wuyishan | E118°0′36″, N27°16′12″ | X8 | X9 |
| WX31 | Wuyishan | E118°0′36″, N27°16′12″ | X9 | X7 |
| WX32 | Wuyishan | E118°0′36″, N27°16′12″ | X9 | X7 |
| WX33 | Wuyishan | E118°0′36″, N27°16′12″ | X9 | X11 |
| WX34 | Wuyishan | E118°0′36″, N27°16′12″ | X9 | X5 |
| WX35 | Wuyishan | E118°0′36″, N27°16′12″ | X8 | X7 |
| WX36 | Wuyishan | E118°0′36″, N27°16′12″ | X9 | X5 |
| WX37 | Wuyishan | E118°0′36″, N27°16′12″ | X8 | X6 |
| WX38 | Wuyishan | E118°0′36″, N27°16′12″ | X9 | X6 |
| WX39 | Wuyishan | E118°0′36″, N27°16′12″ | X8 | X5 |
| *Q. boulengeri* |  |  |  |  |
| lf1 | Lushan | E116°13′19″, N29°40′06″ | F8 | F2 |
| lf2 | Lushan | E116°13′19″, N29°40′06″ | F8 | F3 |
| lf3 | Lushan | E116°13′19″, N29°40′06″ | F9 | F3 |
| lf4 | Lushan | E116°13′19″, N29°40′06″ | F9 | F3 |
| lf5 | Lushan | E116°13′19″, N29°40′06″ | F9 | F2 |
| lf6 | Lushan | E116°13′19″, N29°40′06″ | F17 | F5 |
| lf7 | Lushan | E116°13′19″, N29°40′06″ | F18 | F5 |
| lf8 | Lushan | E116°13′19″, N29°40′06″ | F17 | F5 |
| lf9 | Lushan | E116°13′19″, N29°40′06″ | F18 | F2 |
| gf1 | Longsheng | E109°58′48″, N25°49′12″ | F1 | F1 |
| gf2 | Longsheng | E109°58′48″, N25°49′12″ | F2 | F4 |
| gf3 | Longsheng | E109°58′48″, N25°49′12″ | F4 | F6 |
| gf4 | Longsheng | E109°58′48″, N25°49′12″ | F5 | F6 |
| gf5 | Longsheng | E109°58′48″, N25°49′12″ | F5 | F6 |
| gf6 | Longsheng | E109°58′48″, N25°49′12″ | F6 | F6 |
| gf7 | Longsheng | E109°58′48″, N25°49′12″ | F6 | F4 |
| gf8 | Longsheng | E109°58′48″, N25°49′12″ | F6 | F4 |
| gf9 | Longsheng | E109°58′48″, N25°49′12″ | F6 | F8 |
| gf10 | Longsheng | E109°58′48″, N25°49′12″ | F6 | F1 |
| gf11 | Longsheng | E109°58′48″, N25°49′12″ | F5 | F8 |
| gf12 | Longsheng | E109°58′48″, N25°49′12″ | F6 | F8 |
| gf13 | Longsheng | E109°58′48″, N25°49′12″ | F9 | F8 |
| gf14 | Longsheng | E109°58′48″, N25°49′12″ | F6 | F6 |
| gf15 | Longsheng | E109°58′48″, N25°49′12″ | F22 | F1 |
| gf16 | Longsheng | E109°58′48″, N25°49′12″ | F6 | F1 |
| gf17 | Longsheng | E109°58′48″, N25°49′12″ | F23 | F4 |
| gf18 | Longsheng | E109°58′48″, N25°49′12″ | F12 | F4 |
| gf19 | Longsheng | E109°58′48″, N25°49′12″ | F15 | F1 |
| gf20 | Longsheng | E109°58′48″, N25°49′12″ | F12 | F7 |
| gf21 | Longsheng | E109°58′48″, N25°49′12″ | F3 | F7 |
| gf22 | Longsheng | E109°58′48″, N25°49′12″ | F13 | F8 |
| gf23 | Longsheng | E109°58′48″, N25°49′12″ | F14 | F8 |
| gf24 | Longsheng | E109°58′48″, N25°49′12″ | F16 | F9 |
| gf25 | Longsheng | E109°58′48″, N25°49′12″ | F21 | F9 |
| gf26 | Longsheng | E109°58′48″, N25°49′12″ | F20 | F4 |
| gf27 | Longsheng | E109°58′48″, N25°49′12″ | F6 | F1 |
| gf28 | Longsheng | E109°58′48″, N25°49′12″ | F24 | F6 |
| gf29 | Longsheng | E109°58′48″, N25°49′12″ | F16 | F8 |
| gf30 | Longsheng | E109°58′48″, N25°49′12″ | F15 | F8 |
| gf31 | Longsheng | E109°58′48″, N25°49′12″ | F3 | F9 |
| gf32 | Longsheng | E109°58′48″, N25°49′12″ | F15 | F9 |
| gf33 | Longsheng | E109°58′48″, N25°49′12″ | F6 | F7 |
| gf34 | Longsheng | E109°58′48″, N25°49′12″ | F6 | F7 |
| gf35 | Longsheng | E109°58′48″, N25°49′12″ | F16 | F8 |
| gf36 | Longsheng | E109°58′48″, N25°49′12″ | F5 | F8 |
| gf37 | Longsheng | E109°58′48″, N25°49′12″ | F18 | F9 |
| gf38 | Longsheng | E109°58′48″, N25°49′12″ | F13 | F4 |
| gf39 | Longsheng | E109°58′48″, N25°49′12″ | F10 | F6 |
| gf40 | Longsheng | E109°58′48″, N25°49′12″ | F6 | F7 |
| GF1 | Rongjiang | E108°30′54″, N 25°56′17″ | F3 | F11 |
| GF2 | Rongjiang | E108°30′54″, N 25°56′17″ | F3 | F5 |
| GF3 | Rongjiang | E108°30′54″, N 25°56′17″ | F3 | F6 |
| GF4 | Rongjiang | E108°30′54″, N 25°56′17″ | F3 | F6 |
| GF5 | Rongjiang | E108°30′54″, N 25°56′17″ | F2 | F10 |
| GF6 | Rongjiang | E108°30′54″, N 25°56′17″ | F3 | F11 |
| GF7 | Rongjiang | E108°30′54″, N 25°56′17″ | F2 | F11 |
| GF8 | Rongjiang | E108°30′54″, N 25°56′17″ | F3 | F10 |
| GF9 | Rongjiang | E108°30′54″, N 25°56′17″ | F7 | F11 |
| GF10 | Rongjiang | E108°30′54″, N 25°56′17″ | F8 | F5 |
| GF11 | Rongjiang | E108°30′54″, N 25°56′17″ | F3 | F10 |
| GF12 | Rongjiang | E108°30′54″, N 25°56′17″ | F3 | F10 |
| GF13 | Rongjiang | E108°30′54″, N 25°56′17″ | F5 | F10 |
| GF14 | Rongjiang | E108°30′54″, N 25°56′17″ | F3 | F11 |
| GF15 | Rongjiang | E108°30′54″, N 25°56′17″ | F11 | F11 |
| GF16 | Rongjiang | E108°30′54″, N 25°56′17″ | F8 | F13 |
| GF17 | Rongjiang | E108°30′54″, N 25°56′17″ | F2 | F11 |
| GF18 | Rongjiang | E108°30′54″, N 25°56′17″ | F3 | F12 |
| GF19 | Rongjiang | E108°30′54″, N 25°56′17″ | F8 | F13 |
| GF20 | Rongjiang | E108°30′54″, N 25°56′17″ | F3 | F13 |
| GF21 | Rongjiang | E108°30′54″, N 25°56′17″ | F4 | F11 |
| GF22 | Rongjiang | E108°30′54″, N 25°56′17″ | F1 | F12 |
| GF23 | Rongjiang | E108°30′54″, N 25°56′17″ | F2 | F10 |
| *Q. jiulongensis* |  |  |  |  |
| WL1 | Wuyishan | E118°0′36″, N27°16′12″ | L10 | L2 |
| WL2 | Wuyishan | E118°0′36″, N27°16′12″ | L7 | L2 |
| WL3 | Wuyishan | E118°0′36″, N27°16′12″ | L10 | L4 |
| WL4 | Wuyishan | E118°0′36″, N27°16′12″ | L2 | L1 |
| WL5 | Wuyishan | E118°0′36″, N27°16′12″ | L2 | L4 |
| WL6 | Wuyishan | E118°0′36″, N27°16′12″ | L8 | L4 |
| WL7 | Wuyishan | E118°0′36″, N27°16′12″ | L8 | L4 |
| WL8 | Wuyishan | E118°0′36″, N27°16′12″ | L7 | L4 |
| WL9 | Wuyishan | E118°0′36″, N27°16′12″ | L2 | L2 |
| WL10 | Wuyishan | E118°0′36″, N27°16′12″ | L2 | L2 |
| WL11 | Wuyishan | E118°0′36″, N27°16′12″ | L7 | L1 |
| WL12 | Wuyishan | E118°0′36″, N27°16′12″ | L7 | L1 |
| WL13 | Wuyishan | E118°0′36″, N27°16′12″ | L2 | L1 |
| WL14 | Wuyishan | E118°0′36″, N27°16′12″ | L2 | L2 |
| WL15 | Wuyishan | E118°0′36″, N27°16′12″ | L2 | L2 |
| WL16 | Wuyishan | E118°0′36″, N27°16′12″ | L7 | L4 |
| WL17 | Wuyishan | E118°0′36″, N27°16′12″ | L7 | L4 |
| WL18 | Wuyishan | E118°0′36″, N27°16′12″ | L7 | L2 |
| WL19 | Wuyishan | E118°0′36″, N27°16′12″ | L9 | L2 |
| WL20 | Wuyishan | E118°0′36″, N27°16′12″ | L9 | L1 |
| WL21 | Wuyishan | E118°0′36″, N27°16′12″ | L9 | L1 |
| JL1 | Jiulongshan | E118°53′21″, N28°21′41″ | L1 | L7 |
| JL2 | Jiulongshan | E118°53′21″, N28°21′41″ | L1 | L5 |
| JL3 | Jiulongshan | E118°53′21″, N28°21′41″ | L4 | L5 |
| JL4 | Jiulongshan | E118°53′21″, N28°21′41″ | L4 | L5 |
| JL5 | Jiulongshan | E118°53′21″, N28°21′41″ | L4 | L5 |
| JL6 | Jiulongshan | E118°53′21″, N28°21′41″ | L2 | L3 |
| JL7 | Jiulongshan | E118°53′21″, N28°21′41″ | L2 | L2 |
| JL8 | Jiulongshan | E118°53′21″, N28°21′41″ | L2 | L3 |
| JL9 | Jiulongshan | E118°53′21″, N28°21′41″ | L4 | L3 |
| JL10 | Jiulongshan | E118°53′21″, N28°21′41″ | L5 | L6 |
| JL11 | Jiulongshan | E118°53′21″, N28°21′41″ | L5 | L6 |
| JL12 | Jiulongshan | E118°53′21″, N28°21′41″ | L5 | L5 |
| JL13 | Jiulongshan | E118°53′21″, N28°21′41″ | L5 | L5 |
| JL14 | Jiulongshan | E118°53′21″, N28°21′41″ | L4 | L5 |
| JL15 | Jiulongshan | E118°53′21″, N28°21′41″ | L4 | L5 |
| JL16 | Jiulongshan | E118°53′21″, N28°21′41″ | L2 | L7 |
| JL17 | Jiulongshan | E118°53′21″, N28°21′41″ | L6 | L7 |
| JL18 | Jiulongshan | E118°53′21″, N28°21′41″ | L6 | L6 |
| JL19 | Jiulongshan | E118°53′21″, N28°21′41″ | L6 | L6 |
| JL20 | Jiulongshan | E118°53′21″, N28°21′41″ | L1 | L3 |
| JL21 | Jiulongshan | E118°53′21″, N28°21′41″ | L1 | L3 |
| JL22 | Jiulongshan | E118°53′21″, N28°21′41″ | L3 | L3 |
| JL23 | Jiulongshan | E118°53′21″, N28°21′41″ | L3 | L3 |
| SL1 | Songyang | E119°29′7″, N28°27′23″ | L3 | L6 |
| SL2 | Songyang | E119°29′7″, N28°27′23″ | L3 | L8 |
| SL3 | Songyang | E119°29′7″, N28°27′23″ | L3 | L7 |
| SL4 | Songyang | E119°29′7″, N28°27′23″ | L5 | L7 |
| SL5 | Songyang | E119°29′7″, N28°27′23″ | L5 | L7 |
| SL6 | Songyang | E119°29′7″, N28°27′23″ | L3 | L6 |
| SL7 | Songyang | E119°29′7″, N28°27′23″ | L3 | L8 |
| SL8 | Songyang | E119°29′7″, N28°27′23″ | L3 | L8 |
| SL9 | Songyang | E119°29′7″, N28°27′23″ | L3 | L8 |
| *Q.shini* |  |  |  |  |
| gc1 | Longsheng | E109°58′48″,  N25°49′12″ | C1 | C5 |
| gc2 | Longsheng | E109°58′48″,  N25°49′12″ | C1 | C5 |
| gc3 | Longsheng | E109°58′48″,  N25°49′12″ | C1 | C5 |
| gc4 | Longsheng | E109°58′48″,  N25°49′12″ | C1 | C5 |
| gc5 | Longsheng | E109°58′48″,  N25°49′12″ | C4 | C9 |
| gc6 | Longsheng | E109°58′48″,  N25°49′12″ | C5 | C9 |
| gc7 | Longsheng | E109°58′48″,  N25°49′12″ | C1 | C5 |
| gc8 | Longsheng | E109°58′48″,  N25°49′12″ | C1 | C5 |
| lc1 | Lushan | E116°13′19″,  N29°40′06″ | C2 | C2 |
| lc2 | Lushan | E116°13′19″,  N29°40′06″ | C2 | C2 |
| lc3 | Lushan | E116°13′19″,  N29°40′06″ | C2 | C1 |
| lc4 | Lushan | E116°13′19″,  N29°40′06″ | C3 | C4 |
| lc5 | Lushan | E116°13′19″,  N29°40′06″ | C3 | C1 |
| lc6 | Lushan | E116°13′19″,  N29°40′06″ | C3 | C2 |
| lc7 | Lushan | E116°13′19″,  N29°40′06″ | C3 | C6 |
| lc8 | Lushan | E116°13′19″,  N29°40′06″ | C2 | C9 |
| lc9 | Lushan | E116°13′19″,  N29°40′06″ | C2 | C7 |
| lc10 | Lushan | E116°13′19″,  N29°40′06″ | C2 | C2 |
| lc11 | Lushan | E116°13′19″,  N29°40′06″ | C1 | C2 |
| lc12 | Lushan | E116°13′19″,  N29°40′06″ | C3 | C4 |
| lc13 | Lushan | E116°13′19″,  N29°40′06″ | C3 | C1 |
| lc14 | Lushan | E116°13′19″,  N29°40′06″ | C3 | C2 |
| lc15 | Lushan | E116°13′19″,  N29°40′06″ | C3 | C4 |
| lc16 | Lushan | E116°13′19″,  N29°40′06″ | C3 | C4 |
| lc17 | Lushan | E116°13′19″,  N29°40′06″ | C2 | C3 |
| lc18 | Lushan | E116°13′19″,  N29°40′06″ | C2 | C3 |
| lc19 | Lushan | E116°13′19″,  N29°40′06″ | C2 | C6 |
| lc20 | Lushan | E116°13′19″,  N29°40′06″ | C3 | C6 |
| lc21 | Lushan | E116°13′19″,  N29°40′06″ | C2 | C7 |
| lc22 | Lushan | E116°13′19″,  N29°40′06″ | C2 | C1 |
| lc23 | Lushan | E116°13′19″,  N29°40′06″ | C3 | C4 |
| lc24 | Lushan | E116°13′19″,  N29°40′06″ | C3 | C2 |
